# Study on hydration characteristics and micromechanical properties of illite based on molecular dynamics

**DOI:** 10.1371/journal.pone.0346820

**Published:** 2026-05-12

**Authors:** Mian Li, Zhangping Yan, Yang Xu, Qiuqi Chen, Yuhang Zhou, Xin Tang

**Affiliations:** 1 Chongqing Vocational Institute of Safety Technology, Chongqing City, China; 2 Chongqing Sanxia University of Science and Technology, Chongqing City, China; 3 China University of Mining and Technology, Jiangsu City, China; China University of Mining and Technology, CHINA

## Abstract

This study focuses on the water absorption process of illite, a heavy clay mineral of oil shale, and its related physical, chemical and mechanical properties. The interaction between water and illite molecules under different temperature, pressure and water saturation was studied by coupling molecular simulation. The changes and influence characteristics of basic physical properties (volume, density, expansion effect) and mechanical properties (elastic modulus, Poisson ‘s ratio, mechanical heterogeneity) of hydrated illite were analyzed. The results of molecular simulation show that the water absorption of illite mainly expands in the z direction of the crystal, the pressure can inhibit the expansion, and the temperature and water saturation promote the expansion. The interaction between oxygen atoms and potassium ions in water is more intense under pressure, and temperature and water saturation promote the diffusion of water molecules. The elastic modulus of illite increases with the increase of pressure and decreases with the increase of temperature and water saturation. The research results provide a theoretical basis for the stability evaluation of clay minerals in the fields of petroleum exploitation and geotechnical engineering, and reveal the microscopic mechanism of illite hydration degradation under multi-field coupling conditions.

## Introduction

As a fine-grained porous material, clay minerals are ubiquitous in shale reservoir sediments. Their strong adsorption capacity, high swelling potential, and large specific surface area significantly impact the application of fracturing technologies in shale oil exploitation [1]. Recent molecular dynamics (MD) studies have advanced our understanding of clay hydration mechanisms at the atomic scale: Sun et al [2]. used multi-scale MD simulations to reveal water-induced structural weakening in kaolinite, establishing a direct link between mechanical degradation and heterogeneous pore pressure distributions. While Yang et al [3]. further clarified that Coulombic interactions dominate the swelling behavior of illite-montmorillonite mixed layers through energy decomposition analysis. Complementing these simulation findings, Lu et al [4]. experimentally identified interlayer slippage as the primary mechanism driving softening of red-bed claystones during hydration.

Beyond structural changes, tensile property studies have uncovered clay-specific mechanical responses: Wei et al [5,6]. observed anisotropic failure modes in hydrated montmorillonite via MD-based uniaxial tensile tests, in stark contrast to the water-resistant tensile stability of illite reported by Lu et al [4]. While extensive simulations and experiments have confirmed that water induces deformation and mechanical property degradation in clay minerals, the specific mechanisms by which illite hydration contributes to the overall mechanical failure of shale formations remain incompletely understood. This knowledge gap has motivated further research into the processes and mechanisms governing hydration-induced mechanical degradation in illite-bearing shales.

Wang et al [7]. investigated the evolution of hydro-mechanical properties of clay under different heating durations and humidity exposure times, revealing the microscale mechanisms of hydro-mechanical property deterioration. Their findings indicated that supercritical hydration conditions induce mechanical degradation in clay through synergistic effects of hydration expansion and microstructural damage. Based on the structural characteristics of clay minerals, Zhang et al [8]. systematically discussed the mass transport behavior and mechanical weakening mechanisms of montmorillonite hydration under coupled variations in water content, temperature, and pressure. Their results demonstrated that increasing water content directly correlates with expanded clay interlayer spacing. Furthermore, changes in temperature and pressure significantly influence the diffusion of interlayer Na⁺ during hydration, thereby modulating the extent of clay mechanical degradation.

Complementing these experimental studies, Li et al [9]. employed molecular simulations to investigate the microstructural evolution and mechanical properties of hydrated clay under extreme high-temperature and high-pressure conditions. Their simulations revealed contrasting effects of temperature and pressure on clay mass transport and mechanical performance: elevated temperature facilitated the diffusion of interlayer water molecules and Na^+^, accelerating the rate of mechanical property degradation in clay minerals

Mounting evidence indicates that montmorillonite undergoes illitization under natural geological conditions, including high temperature, high pressure, and aqueous environments, with profound implications for the overall structure and mechanical properties of shale formations [10]. Beyond mineralogical transformations, Jiang et al [11]. established a direct link between illite occurrence in geomaterials and geological engineering hazards. Their research revealed that the differential swelling behavior between illite and detrital minerals drives disintegration and structural failure in rock-soil systems. This differential expansion creates pervasive disintegration cracks, which further reduce the safety factor of slopes and increase the likelihood of catastrophic geohazards. The temperature-pressure-saturation (T-P-Saturation) full coverage scanning of the system, by using the Cv index to explicitly quantify the mechanical heterogeneity, some scholars [12] showed that molecular dynamics (MD) simulation can reveal the adsorption configuration, diffusion behavior and intermolecular interaction of gas-liquid-solid surfactants at the atomic scale, thus providing insights that cannot be obtained by experiments alone [13].

The research described in this paper is compared with the previous MD study of illite hydration. By carrying out molecular simulation of hydrated illite under different temperature, pressure and water saturation, the hydration process of clay matrix is deeply understood, and the morphological characteristics and mechanical property degradation mechanism of clay under hydration are revealed. It can better explain his dispersion, which is a key index to evaluate the induced weakening, and provides data support and model verification for the study of shale micromechanics and the deterioration mechanism in the fracturing process of shale oil exploitation. The proposed mechanical inhomogeneity measure provides a quantitative relationship between molecular-scale hydration behavior and macro-scale wellbore instability, enabling engineers to predict wellbore collapse caused by clay expansion by integrating atomic simulation data into geomechanical models.

### Simulation details

#### Model construction.

**Fig 1:**
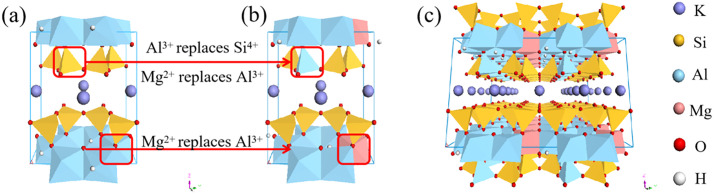
Construction process of illite model. **(a)** Illite original cell model; **(b)** Illite cell model after ion replacement; **(c)** Illite supercell model.

Illite is a kind of mica. From the microscopic point of view, its structure is a relatively regular crystal cell structure, which is rich in potassium, aluminum, silicon, oxygen, hydrogen and other elements. Bish et al [14] used XRD experiments to test and analyze the crystal cell structure of illite, and obtained the lattice parameters of illite cell and the spatial information of each element in the cell. The unit cell space group of illite is C2/ m. The lattice sizes a, b and c are 5.2021 Å, 8.9797 Å and 10.2260 Å, respectively. The lattice angles α, β and γ are 90°, 101.57° and 90°, respectively. Based on the Cartesian coordinate information of each element in the unit cell, the illite single cell model (Fig 1a) was constructed. Based on the Lowenstein substitution principle, the elements in the illite unit cell were replaced by ions (Fig 1b), and the supercellization of 4a × 2b × 1c was carried out to construct the illite supercell model (Fig 1c). In a reasonable range of water saturation, increasing the water content according to the gradient is conducive to the analysis of the basic properties of illite under each water saturation. The water content is 0,9,18,27,36 and 45, respectively, to construct six groups of hydrated illite models with different water saturation (Table 1). Illite is a TOT structure, and the constructed water molecules are directly added to the interlayer of illite. The water saturation is the ratio of the mass of the water film to the mass of the single crystal surface in the water-bearing crystal. In this paper, it is the ratio of the mass of water molecules to the mass of a layer of illite.

**Table 1:** Illite water saturation under different hydration degree.

Number of water molecules	0	9	18	27	36	45
**Water saturation/ %**	0.00	5.098	10.197	15.295	20.394	25.492

### Simulation system and force field

**Fig 2:**
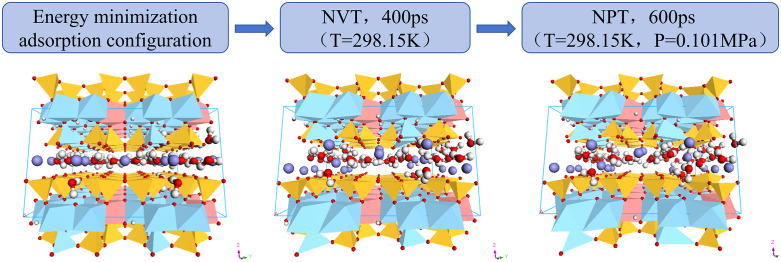
Molecular dynamics simulation process.

In the molecular dynamics simulation, the three-dimensional periodic structure is used to construct the illite crystal, so that it exhibits three-way evolution under different temperature and pressure environments to conform to the actual situation. The structure was optimized and the temperature of illite system was controlled by molecular dynamics simulation under Canonical Ensemble (NVT) ensemble. The last frame configuration was selected and molecular dynamics simulation was carried out under Isothermal-isobaric Ensemble (NPT) ensemble to explore the physical and chemical properties of illite under different pressures [6] (Fig 2). NPT is an anisotropic ensemble, and the size of the simulation box a, b, c) is allowed to fluctuate independently to ensure that the internal stress tensor of the system matches the target external pressure. The applied pressure represents the real fluid static pressure, and the uniform pressure is applied in the three spindle directions. This setting simulates the situation of unconstrained expansion or the environment of fractured clay matrix in deep reservoirs [15,16].

We performed a stability check by monitoring the total energy drift under the NVE ensemble to prove the rationality of using the 1 fs step size at high temperature (200 °C). Specifically, the illite model relaxes 400,000 steps in the NVT ensemble with a step size of 1.0 fs and a total simulation time of 400 ps, while relaxes 600,000 steps in the NPT ensemble with a step size of 1.0 fs and a total simulation time of 600 ps. In the process of molecular dynamics simulation, when the temperature, total potential energy or density of the simulation system are in a small range for a long time, it is considered that illite reaches a relaxation equilibrium state under this temperature and pressure condition. Among them, the potential function adopts Clayff force field, which is suitable for simulating hydration and multi-component mineral systems and their interfaces with aqueous solutions, and is widely used in molecular dynamics simulation of clay. ClayFF is mainly a non-bonding force field, but the resonant bond stretching and bond angle bending terms are only specifically applied to hydroxyl (OH) and water molecules in the illite structure. Other atomic interactions in illite (such as Si-O, Al-O, K-O) follow the ClayFF standard and are treated by Lennard-Jones and Coulomb terms. Under the Clayff force field, the system interaction mainly includes Coulomb force, van der Waals force, bond length stretching potential energy and bond angle distortion potential energy. The calculation method is as follows (Formula 1):


$$\begin{array}{cc} E_{total} & =E_{bond\;stretch}+E_{Coulomb}+E_{VDW}\\ & =k_{\text{1}}{\left(r_{ij}-r_{\text{0}}\right)}^{\text{2}}+\frac{e^{\text{2}}q_{i}q_{j}}{\text{4}\pi \varepsilon_{\text{0}}r_{ij}}+\text{4}\varepsilon_{ij}\left[{\left(\frac{\sigma_{ij}}{r_{ij}}\right)}^{\text{1}\text{2}}-{\left(\frac{\sigma_{ij}}{r_{ij}}\right)}^{\text{6}}\right] \end{array}$$ (1)

where $k_{\text{1}}$ is the force constant, $r_{\text{0}}$ is the equilibrium bond length; $r_{ij}$ is the distance between atoms i and j; $q_{i}$ and $q_{j}$ are the charges of atoms i and j, respectively; $\varepsilon_{\text{0}}$ is the dielectric constant; σ and ε are size and energy parameters, respectively.

In this study, the Water molecular force field model (SPC/ E) is used, which has a good matching with the Clay Force Field (ClayFF) force field and can accurately describe the hydration characteristics of clay minerals. The Particle-Particle Particle-Mesh (PPPM) method is used to calculate the long-range electrostatic interaction, and the calculation accuracy is set to 10^−4^. The truncation distance of Coulomb force and van der Waals force is set to 12.0 Å. The SHAKE algorithm is used to constrain the water molecular structure to maintain its rigidity. Corresponding to different hydration illite, the temperature was set to 25 °C, 50 °C, 100 °C, 200 °C, a total of four groups; the pressures were set to 0.101 MPa, 100 MPa, 200 MPa, 300 MPa, 400 MPa, and 500 MPa, respectively, for a total of six groups. All of the above molecular dynamics simulations are performed by lammps software, which has great functional advantages in computing speed and application range [17,18].

**Fig 3:**
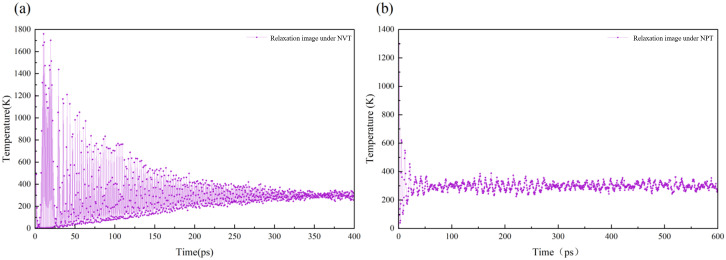
Temperature relaxation diagram under molecular dynamics; (a) The relaxation process under NVT; (b) Relaxation process under NPT.

In the molecular dynamics simulation, with the displacement of each particle in the illite crystal to a relatively reasonable position, the system energy gradually stabilized. When the relaxation time reaches 345 ps, the system temperature fluctuates slightly around 300 K in the molecular dynamics simulation under the NVT ensemble. Therefore, a frame configuration after 345 ps can be selected for subsequent simulation and analysis (Fig 3a). In this study, the last frame configuration after relaxation equilibrium was selected as the research object, and the molecular dynamics simulation and analysis under the subsequent NPT ensemble were carried out. The NPT was used to set the same temperature in the NVT ensemble under the system, and then the pressure was controlled. During the whole molecular dynamics simulation process, when the relaxation time reached 290 ps, the hydrated illite basically reached the relaxation equilibrium at this temperature and pressure (Fig 3b). In the subsequent analysis, instead of analyzing the trajectory file of the whole process of the whole model, the most stable 301–601 frame snapshot of molecular configuration is selected as the research object, and the crystal structure characteristics, expansibility, dynamic performance and basic mechanical properties of hydrated illite in stable state are calculated and analyzed.

### Simulation results and analysis

#### Illite swells by water absorption.

**Fig 4:**
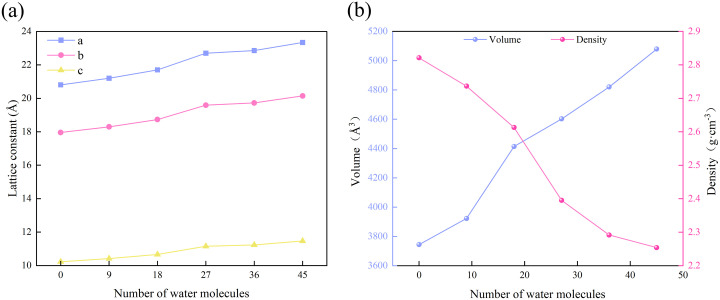
Illite crystal structure parameters under different hydration degree. **(a)** Lattice length parameter; **(b)** Density and volume parameters.

With the increase of water saturation, illite expands, which is mainly reflected in the cell volume, resulting in the change of clay density. Based on the results of molecular dynamics simulation, the lattice length of illite under all frames under different hydration degrees was calculated. On this basis, the changes of volume and density of illite were analyzed. The results show that with the increase of hydration degree, the lattice constant of illite increases gradually, and the lattice expansion rate in the z direction is the most obvious (Fig 4a), which leads to the rapid expansion of the system volume. Although the degree of hydration increases and the mass of the system continues to expand, this is far less than the rate of volume expansion, resulting in a gradual decrease in the density of the system of illite crystal cells (Fig 4b).

The most obvious feature of hydration expansion is the change of interlayer spacing. The interlayer spacing is the spacing between adjacent units of mineral crystal structure in the z direction, which is related to its water saturation and the temperature and pressure conditions of the environment, which will affect the interaction between crystals and crystals and mechanical properties. Through molecular dynamics simulation, the statistical average volume of all frames of the simulated configuration and the statistical average angle of the lattice are analyzed. On this basis, the interlayer spacing of minerals under different conditions can be calculated [19].


$$d_{\text{0}\text{0}\text{1}}=\frac{<V>}{<a><b>sin<\alpha >}$$ (2)

In the formula: <V> is the ensemble statistical average volume of the simulated unit cell; <a>, <b> and <α> are the statistical average values of the corresponding cell parameters, respectively.

**Fig 5:**
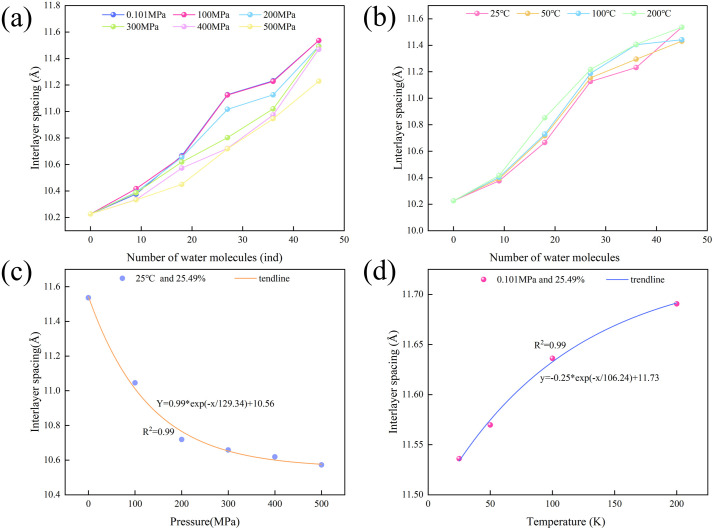
Evolution law of illite basic layer spacing in different environments. **(a)** The evolution of interlayer spacing of hydrated illite at 25 °C; **(b)** The evolution of interlayer spacing of hydrated illite at 0.101 MPa; **(c)** The effect of pressure on interlayer spacing; **(d)** The effect of temperature on interlayer spacing.

When illite is initially immersed in water, interlayer spacing exhibits negligible changes. As hydration progresses, the growth rate of illite interlayer spacing accelerates (Figs 5a and 5b). Water content, temperature, and pressure collectively modulate the evolution of illite interlayer spacing: Under increasing system pressure, illite experiences compression from elevated hydrostatic pressure, which inhibits the hydration expansion process. Consequently, during advanced hydration stages, the increment in interlayer spacing remains minimal (Fig 5a). In contrast, rising system temperature enhances both the overall system energy and interparticle interactions, intensifying molecular thermal motion. This phenomenon facilitates the diffusion of water molecules and interlayer ions, thereby promoting interlayer spacing expansion [20].

Based on this conclusion, the influence of pressure, temperature and water saturation on interlayer spacing is further analyzed. The interlayer spacing of illite with a water saturation of 25.49% decreases with the increase of pressure at a fixed temperature of 25 °C.When the hydrostatic pressure increases from 0.101 MPa to 500 MPa, the particle spacing between the hydrated illite crystals decreases, resulting in a gradual decrease in the interlayer spacing from 11.536 Å to 10.572 Å. The compression rate in the z direction is 8.36%, and the compression coefficient is 1.928 × 10^−3^ Å/ MPa. The interlayer spacing reduction rate also gradually decreases as the internal space of the crystal is gradually compressed and saturated. It shows the evolution trend of compression rate from fast to low (Fig 5c). When the temperature rises from 25 °C to 200 °C, the interlayer spacing of hydrated illite with water saturation of 25.49% increases from 11.537 Å to 11.691 Å, the expansion rate in the z direction is 1.33%, and the expansion rate is 0.7 × 10^−3^ Å/ K. Under the same water saturation, the relationship between illite interlayer spacing and temperature is also non-linear, and its evolution law is opposite to that of illite interlayer spacing under pressure. Temperature promotes the increase of interlayer spacing. With the increase of temperature, the expansion of illite interlayer also shows a trend of rapid and slow evolution (Fig 5d). However, in general, the change of interlayer spacing is still dominated by water saturation. With the increase of hydration degree, the interlayer spacing of illite molecules gradually increases.

### Hydration mechanism of illite

In the nanoscale microscopic field of view, the interaction potential between particles will lead to different acceleration, velocity and spatial coordinate positions of each particle at each moment. This interaction is usually affected by temperature, pressure and the number and type of particles in the system, and is embodied in the arrangement and orientation in its spatial position.

### Radial distribution function

The radial distribution function (RDF) is an index used to quantify the interaction strength between particles. It is expressed as the probability of the occurrence of particle B in the range of r→r+dr centered on the centroid of a particle A, which can reflect the degree of particle aggregation in a certain area, so as to reflect the interaction strength between particle A and particle B. The calculation method is as follows:


$$g(r{)}_{\alpha \beta }=\frac{n(r{)}_{\beta }}{\rho_{\beta }V}\approx \frac{n_{\beta }}{\text{4}\pi \rho_{\beta }r^{\text{2}}dr}$$ (3)

In the formula, $g(r{)}_{\alpha \beta }$ is the distribution probability of particles in a certain region of space, where $n(r{)}_{\beta }$ is the average number of particles j around i in a spherical shell with a radius of r to r+dr. $\rho_{\beta }$ is the number density of particle β. r is the distance between particles i and j, and α is the three-dimensional space volume from particle V in r to r+dr.

**Fig 6:**
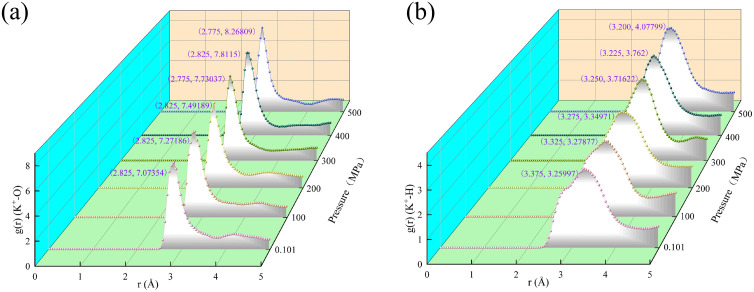
Radial distribution function of hydrated illite with water saturation of 25.39% at 25 °C. **(a)** Radial distribution function of potassium ions and oxygen atoms in water; **(b)** Radial distribution function of potassium ion and hydrogen atom in water.

Under certain conditions, with the increase of system pressure, the peak position of the radial distribution function of potassium ions with oxygen atoms and hydrogen atoms in water shifted. When the pressure increased from 0.101 MPa to 500 MPa, the peak positions of potassium ions and oxygen atoms in water shifted from 2.825 Å to 2.775 Å, from 7.07354 to 8.26809 (Fig 6a). Similarly, the highest peak positions of potassium ions and hydrogen atoms in water are shifted from 3.375 Å to 3.200 Å, respectively, from 3.25997 to 4.07799 (Fig 6b).

**Fig 7:**
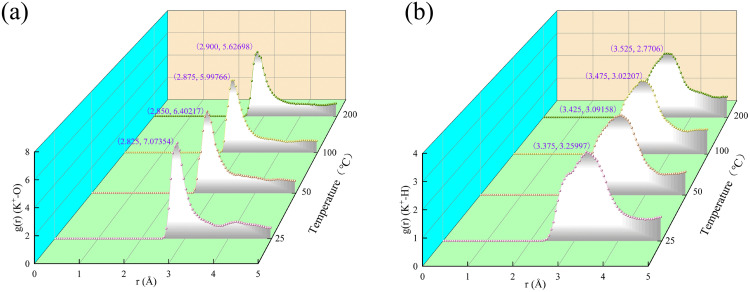
Radial distribution function of hydrated illite with water saturation of 25.39% at 0.101 MPa. **(a)** Radial distribution function of potassium ions and oxygen atoms in water; **(b)** Radial distribution function of potassium ion and hydrogen atom in water.

As the temperature of the system increases, the peak position of the radial distribution function of potassium ions and oxygen atoms and hydrogen atoms in water also shifts as the pressure changes. When the temperature rises from 25 °C to 200 °C, the highest peak position of potassium ions and oxygen atoms in water shifts from 2.825 Å to 2.900 Å, and the highest peak decreases from 7.07354 to 5.62698 (Fig 7a). Similarly, the highest peak position of potassium ion and hydrogen atom in water shifted from 3.375 Å to 3.525 Å, and the highest peak decreased from 3.25997 to 2.7706 (Fig 7b).

**Fig 8:**
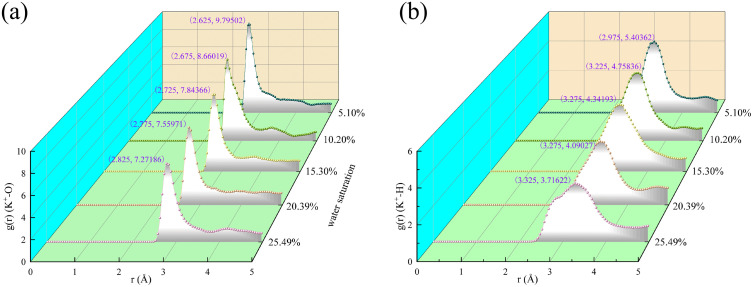
Radial distribution function of hydrated illite with different water saturation at 25 °C and 0.101 MPa. **(a)** Radial distribution function of potassium ions and oxygen atoms in water; **(b)** Radial distribution function of potassium ion and hydrogen atom in water.

When the water saturation of hydrated illite increases from 5.10% to 25.49%, the peak position of potassium ion and oxygen atom in water shifts from 2.625 Å to 2.825 Å, and the peak decreases from 9.79502 to 7.27186 (Fig 8a). Similarly, the highest peak position of potassium ion and hydrogen atom in water shifted from 2.975 Å to 3.325 Å, and the highest peak decreased from 5.40362 to 3.71622 (Fig 8b).

Overall, pressure contributes to the interaction between water molecules and potassium ions between illite interlayers, making them closer in distance; in addition, the peak value of the highest peak also increases with the increase of the system pressure, indicating that the pressure enhances the interaction strength between the hydrogen and oxygen atoms and the potassium ion, and the two are positively correlated in the change trend. Temperature and water saturation are not conducive to the interaction between water molecules and illite interlayer potassium ions, and the temperature rise makes it farther in the interaction distance. In addition, the peak value of the highest peak also decreases with the increase of the system temperature, indicating that the temperature rise inhibits the interaction strength between the hydrogen and oxygen atoms and the potassium ions, and the two are negatively correlated in the trend of change.

### Hydration characteristics

The hydration number describes the arrangement of water molecules around ions. The ion hydration number is an important indicator to truly reflect the degree of hydration of illite, reflecting the binding ability of K ^+^ between illite layers to water molecules. The larger the hydration number, the more stable the hydrated ion structure formed by K ^+^ and water molecules, which makes it difficult for water molecules to penetrate the surface of illite crystals, and then it is difficult to erode the illite lamellae, which can reduce the deterioration effect of illite structure and mechanical properties to a certain extent. Based on the hydration coordination number, the calculation method of ion hydration number is as follows:


$$n_{hyd}={CN}_{\alpha \beta }h$$ (4)

In the formula, ${\text{CN}}_{\alpha \beta }$ is the ion coordination number, h is the hydration factor, and the value of K ^+^ hydration factor is 0.4 [21].

The hydration radius provides the scale information of the interaction between water molecules and ions, which is helpful to judge the thickness and effectiveness of the hydration layer. By analyzing the change of hydration radius, it can be seen that the hydration radius is the range of action of inorganic cations on water molecules, which is shown as the radius of hydrated ion structure sphere wrapped by inorganic cations. It can be used to analyze the size and compactness of water shell around cations [22]. In order to quantitatively analyze the degree of hydration shell convergence of hydrated potassium ions between illite layers and explore the hydration strength of illite under different water saturations, the hydration radius should be calculated:


$$\frac{\text{4}}{\text{3}}\pi \overset{\text{3}}{\underset{hyd}{r}}=V_{H_{\text{2}}O}n_{hyd}+\frac{\text{4}}{\text{3}}\pi \overset{\text{3}}{\underset{eff}{r}}$$ (5)

In the formula, $r_{\text{hyd}}$ is the hydration radius, ${\text{V}}_{{\text{H}}_{\text{2}}\text{O}}$ is the volume of water molecules, the value is 0.02991 nm^3^, $r_{\text{eef}}$ is the effective radius of ions, $g(r{)}_{\alpha \beta }$ is the difference between the effective radius of the first peak corresponding to abscissa r and water molecules, here the value is the effective radius of water molecules, 0.138 nm [23].

**Fig 9:**
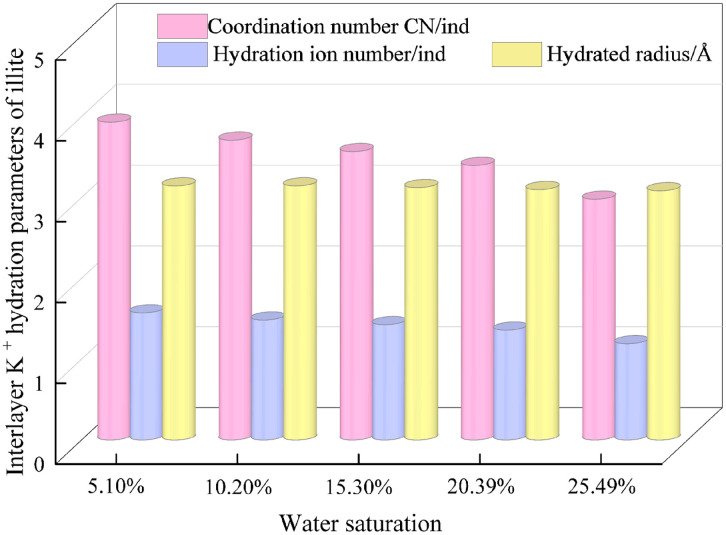
K ^+^ hydration parameters of illite interlayer.

Through the calculation of hydration parameters, the K ^+^ hydration parameters of illite interlayer under different water saturation were obtained. When the water saturation of hydrated illite increases from 5.10% to 25.49%, the ion coordination number and hydration number increase with the decrease of water saturation, indicating that K ^+^ is more closely combined with the surrounding water molecules at lower water saturation. The smaller the radius of the hydrated potassium ion sphere is, the smaller the hydration radius is. At this time, the diffusivity of K ^+^ and interlayer water is weak in the hydrated illite system [24]. At higher water saturation, K ^+^ is surrounded by more water molecules, and the hydrated potassium ion structure formed by K ^+^ and surrounding water molecules becomes gradually unstable, and the sparse hydrated structure shows an increasing trend in its hydration radius (Fig 9).

The hydration degree of illite can be reflected by hydration parameters. The hydration process of illite by water molecules is mainly reflected in the process of water molecules being adsorbed by interlayer K ^+^. The increasing water saturation will increase the hydrated ion water between the two and gradually occupy the limited K ^+^ adsorption site. When the hydration capacity of K ^+^ is fully used, the excess water molecules will gradually erode the crystal lamellae along the 001 structural plane of illite crystal, destroy the crystal structure and stability of illite, and cause the deterioration of its mechanical properties. Therefore, the interlayer cations have the properties of adsorbing water molecules. Under certain conditions, high concentrations of inorganic salt components are added to the minerals, which provides new possibilities for inhibiting the erosion and damage of clay minerals represented by illite in the water environment.

### Dynamic diffusion characteristics

In molecular dynamics simulation, particle diffusivity is commonly expressed by diffusion coefficient. Diffusion coefficient (D) is calculated by the root mean square displacement of particles, which can show the degree of migration of particles over time, and show the characteristic attribute of particle dynamics. The calculation method is as follows:


$$\begin{array}{c} D=\frac{\text{1}}{\text{6}N_{\alpha }}\underset{t\to \infty }{lim}\frac{d}{dt}\sum \overset{N_{\alpha }}{\underset{i=\text{1}}{\mathrm{\backslash nolimits}}}\{[\begin{array}{c} r_{i}(t)-r_{i}(\text{0}) \end{array}]\text{2}\} \end{array}$$ (6)

In the formula, $[\begin{array}{c} r_{i}(t)-r_{i}(\text{0}) \end{array}]\text{2}$ refers to the root mean square displacement of particle i, where, $r_{i}(\text{0})$ is the position of particle i at time 0, and $r_{i}(t)$ is the position of particle i at time t. $N_{\alpha }$ is the total number of particles α.

**Fig 10:**
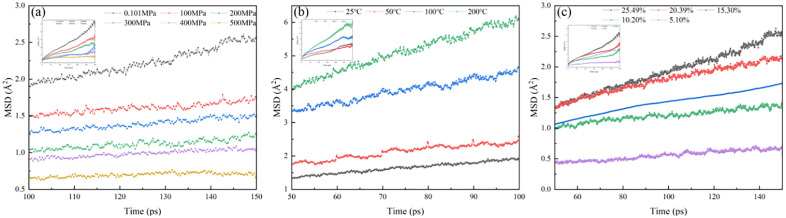
Mean square displacement of water molecules between illite layers. **(a)** Mean square displacement of water molecules under pressure; **(b)** Mean square displacement of water molecules under temperature; **(c)** Mean square displacement of water molecules under different water saturations.

At 25 °C, as the system pressure increases from 0.101 MPa to 500 MPa, the interlayer water diffusion coefficient of illite with water saturation of 25.39% decreases from 0.223 × 10^−10^ m^2^/ s to 0.015 × 10^−10^ m^2^/ s (Fig 10a). When the system pressure is fixed at 0.101 MPa, with the increase of temperature from 25 °C to 200 °C, the interlayer water diffusion coefficient of illite with water saturation of 25.39% increases from 0.228 × 10^−10^ m^2^/ s to 0.682 × 10^−10^ m^2^/ s (Fig 10b). Under the temperature and pressure conditions of 25 °C and 0.101 MPa, with the increase of illite water saturation from 5.10% to 25.39%, the interlayer water diffusion coefficient of illite increases from 0.043 to 0.195 (Fig 10c).

It can be seen that with the increase of pressure, the diffusivity of interlayer water in illite gradually decreases. Within a certain range, the increase of system pressure can effectively control the migration of water molecules. Due to the increase of temperature, the energy of the hydrated illite system becomes higher and unstable, and the water molecules obtain greater kinetic energy, which aggravates the irregular movement of water molecules and has strong diffusivity. The content of water molecules in the system continues to increase, the occurrence space is gradually occupied, and the limited potassium ions between illite layers cannot adsorb a large amount of water molecules in a coordinated manner. Therefore, water molecules will spontaneously migrate to low concentration areas, showing strong diffusivity with the increase of water saturation.

### Mechanical properties characteristics

In molecular dynamics simulation, the calculation of elastic modulus is based on Hooke ‘s law. By calculating the stress and strain of the crystal, the stiffness matrix ${\text{C}}_{\text{ij}}$ and the flexibility matrix ${\text{S}}_{\text{ij}}$ are analyzed. According to Voigt-Reuss theory, the matrix is simplified to obtain the upper limit and lower limit of elastic modulus. Finally, the arithmetic mean of elastic modulus is obtained based on Hil theory. According to Voigt-Reuss-Hill theory, the matrix is simplified and calculated, and the stiffness matrix and flexibility matrix of 6 × 6 are obtained respectively.

The stiffness matrix ${\text{ C}}_{\text{ij}}$ is denoted by equation 7:


$$\left[\begin{array}{cccccc} {\text{C}}_{\text{11}} & {\text{C}}_{\text{12}} & {\text{C}}_{\text{13}} & {\text{C}}_{\text{14}} & {\text{C}}_{\text{15}} & {\text{C}}_{\text{16}}\\ {\text{C}}_{\text{21}} & {\text{C}}_{\text{22}} & {\text{C}}_{\text{23}} & {\text{C}}_{\text{24}} & {\text{C}}_{\text{25}} & {\text{C}}_{\text{26}}\\ {\text{C}}_{\text{31}} & {\text{C}}_{\text{32}} & {\text{C}}_{\text{33}} & {\text{C}}_{\text{34}} & {\text{C}}_{\text{35}} & {\text{C}}_{\text{36}}\\ {\text{C}}_{\text{41}} & {\text{C}}_{\text{42}} & {\text{C}}_{\text{43}} & {\text{C}}_{\text{44}} & {\text{C}}_{\text{45}} & {\text{C}}_{\text{46}}\\ {\text{C}}_{\text{51}} & {\text{C}}_{\text{52}} & {\text{C}}_{\text{53}} & {\text{C}}_{\text{54}} & {\text{C}}_{\text{55}} & {\text{C}}_{\text{56}}\\ {\text{C}}_{\text{61}} & {\text{C}}_{\text{62}} & {\text{C}}_{\text{63}} & {\text{C}}_{\text{64}} & {\text{C}}_{\text{65}} & {\text{C}}_{\text{66}} \end{array}\right]$$ (7)

The flexibility matrix ${\text{S}}_{\text{ij}}$ is denoted by equation 8:


$$\left[\begin{array}{cccccc} {\text{S}}_{\text{11}} & {\text{S}}_{\text{12}} & {\text{S}}_{\text{13}} & {\text{S}}_{\text{14}} & {\text{S}}_{\text{15}} & {\text{S}}_{\text{16}}\\ {\text{S}}_{\text{21}} & {\text{S}}_{\text{22}} & {\text{S}}_{\text{23}} & {\text{S}}_{\text{24}} & {\text{S}}_{\text{25}} & {\text{S}}_{\text{26}}\\ {\text{S}}_{\text{31}} & {\text{S}}_{\text{32}} & {\text{S}}_{\text{33}} & {\text{S}}_{\text{34}} & {\text{S}}_{\text{35}} & {\text{S}}_{\text{36}}\\ {\text{S}}_{\text{41}} & {\text{S}}_{\text{42}} & {\text{S}}_{\text{43}} & {\text{S}}_{\text{44}} & {\text{S}}_{\text{45}} & {\text{S}}_{\text{46}}\\ {\text{S}}_{\text{51}} & {\text{S}}_{\text{52}} & {\text{S}}_{\text{53}} & {\text{S}}_{\text{54}} & {\text{S}}_{\text{55}} & {\text{S}}_{\text{56}}\\ {\text{S}}_{\text{61}} & {\text{S}}_{\text{62}} & {\text{S}}_{\text{63}} & {\text{S}}_{\text{64}} & {\text{S}}_{\text{65}} & {\text{S}}_{\text{66}} \end{array}\right]$$ (8)

For the elastic modulus, the Voigt model calculates the upper limit elastic modulus of the material (i.e., the maximum value of its elastic modulus). The bulk modulus (${\text{B}}_{\text{V}}$) and shear modulus (${\text{G}}_{\text{V}}$) of the polycrystal can be calculated by the stiffness matrix:


$$B_{V}=\frac{\left(C_{\text{1}\text{1}}+C_{\text{2}\text{2}}+C_{\text{3}\text{3}}\right)+\text{2}\left(C_{\text{1}\text{2}}+C_{\text{1}\text{3}}+C_{\text{2}\text{3}}\right)}{\text{9}}$$ (9)

$${\text{G}}_{\text{V}}=\frac{\left({\text{C}}_{\text{11}}+{\text{C}}_{\text{22}}+{\text{C}}_{\text{33}}\right)-\left({\text{C}}_{\text{12}}+{\text{C}}_{\text{13}}+{\text{C}}_{\text{23}}\right)+\text{3}\left({\text{C}}_{\text{44}}+{\text{C}}_{\text{55}}+{\text{C}}_{\text{66}}\right)}{\text{15}}$$ (10)

On the contrary, the Reuss model assumes that the stress applied to the material is the same, so the strain of the material is calculated by the average of each grain. This model calculates the lower bound elastic modulus of the material (i.e., the minimum value of its elastic modulus). The bulk modulus (${\text{B}}_{\text{R}}$) and shear modulus (${\text{G}}_{\text{R}}$) of the polycrystal can be calculated by the stiffness matrix:


$$B_{R}=\frac{\text{1}}{\left(S_{\text{1}\text{1}}+S_{\text{2}\text{2}}+S_{\text{3}\text{3}}\right)+\text{2}\left(S_{\text{1}\text{2}}+S_{\text{1}\text{3}}+S_{\text{2}\text{3}}\right)}$$ (11)

$$G_{R}=\frac{\text{1}}{\text{4}\left(S_{\text{1}\text{1}}+S_{\text{2}\text{2}}+S_{\text{3}\text{3}}\right)-\text{4}\left(S_{\text{1}\text{2}}+S_{\text{1}\text{3}}+S_{\text{2}\text{3}}\right)+\text{3}\left(S_{\text{4}\text{4}}+S_{\text{5}\text{5}}+S_{\text{6}\text{6}}\right)}$$ (12)

$$B_{H}=\frac{B_{V}+B_{R}}{\text{2}}$$ (13)

$$G_{H}=\frac{G_{V}+G_{R}}{\text{2}}$$ (14)

The Young ‘s modulus (${\text{B}}_{\text{H}}$) and Poisson ‘s ratio (${\text{G}}_{\text{H}}$) of polycrystals can be deduced from the bulk modulus (E) and shear modulus (ν) calculated by Hill ‘s theory:


$$E=\frac{\text{9}B_{H}G_{H}}{\text{3}B_{H}+G_{H}}$$ (15)

$$\nu =\frac{\text{3}{\text{B}}_{\text{H}}-\text{2}{\text{G}}_{\text{H}}}{\text{2}\left(\text{3}{\text{B}}_{\text{H}}+{\text{G}}_{\text{H}}\right)}$$ （16)

### Mechanical properties of hydrated illite

**Fig 11:**
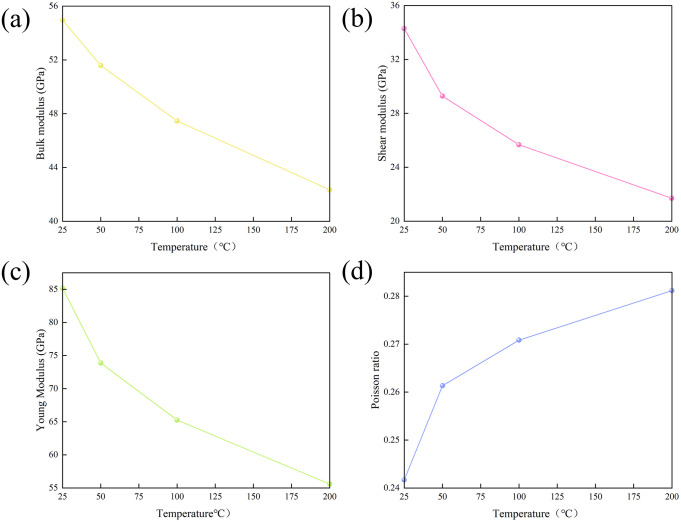
Mechanism of temperature effect on mechanical properties. **(a)** bulk modulus; **(b)** Shear modulus; **(c)** Young ‘s modulus; **(d)** Poisson ‘s ratio.

At 0.101 MPa, the elastic modulus of hydrated illite with water saturation of 25.49% decreases with the increase of temperature. When the temperature increases from 25 °C to 200 °C, the attenuation range of bulk modulus, shear modulus and Young ‘s modulus is 54.958 - 42.339 GPa, 34.299 - 21.696 GPa and 85.178 - 55.591 GPa, respectively, with a decrease of 12.620 GPa, 12.603 GPa and 29.586 GPa, respectively (Figs 11a - 11c). In contrast, the Poisson ‘s ratio increased from 0.242 to 0.281, an increase of 0.039 (Fig 11d).

With the increase of temperature, the atomic movement in the hydrated illite intensifies, resulting in the thermal expansion of the lattice and the increase of the atomic spacing. For hydrated illite, the increased atomic spacing may reduce its overall rigidity. At the same time, the behavior of potassium hydrated water molecules changes with temperature under high temperature conditions, which has a direct impact on the mechanical properties of the mineral. The bulk modulus, shear modulus and Young ‘s modulus decrease with the increase of temperature, and the compressibility increases. Within a certain range, hydrated illite is more likely to be deformed by temperature and stress. In addition, the Poisson ‘s ratio of hydrated illite is positively correlated with the evolution of temperature, reflecting that the transverse deformation of hydrated illite is more significant under the action of axial stress.

**Fig 12:**
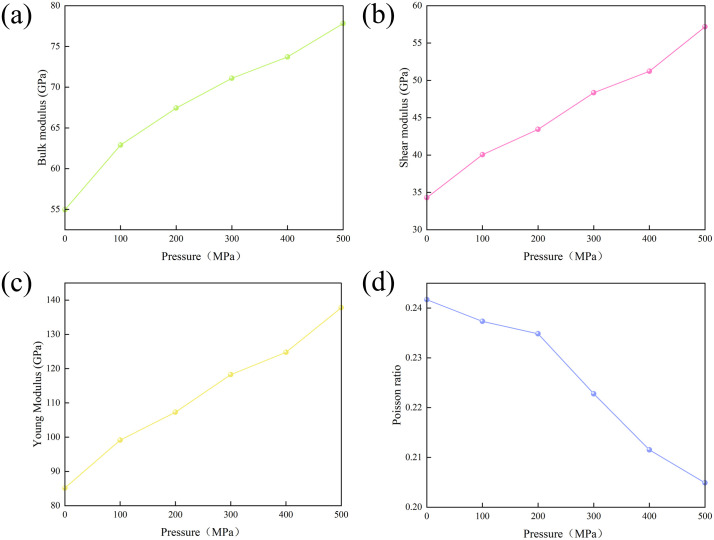
Mechanism of pressure effect on mechanical properties. **(a)** bulk modulus; **(b)** Shear modulus; **(c)** Young ‘s modulus; **(d)** Poisson ‘s ratio.

When the system pressure increases from 0.101 MPa to 500 MPa, the bulk modulus, shear modulus, and Young ‘s modulus increase in the range of 54.958 - 77.820 GPa, 34.299-57.181 GPa, and 85.178 - 137.792 GPa, respectively, with an increase of 22.861 GPa, 22.882 GPa, and 52.615 GPa, respectively (Figs 12a - 12c). In contrast, the Poisson ‘s ratio decreased from 0.242 to 0.205, a decrease of 0.037 (Fig 12d)

Contrary to the mechanism of temperature action, increasing the pressure will lead to an increase in the interaction force between the atoms in the hydrated illite, thereby reducing the atomic spacing and enhancing the compactness of the hydrated illite [25]. The high pressure environment will affect the mechanical behavior of potassium water molecules and enhance the interaction between water molecules. The bulk modulus, shear modulus and Young ‘s modulus increase with the increase of pressure, reflecting that the compressive capacity of the material is enhanced, and the deformation capacity under stress is reduced. The overall mechanical strength of hydrated illite is positively correlated with pressure. It is negatively correlated with the evolution law of pressure, and the change range is slightly smaller than the Poisson ‘s ratio change under the action of temperature, reflecting that within a certain range, the transverse deformation of hydrated illite will be smaller under the action of axial stress.

**Fig 13:**
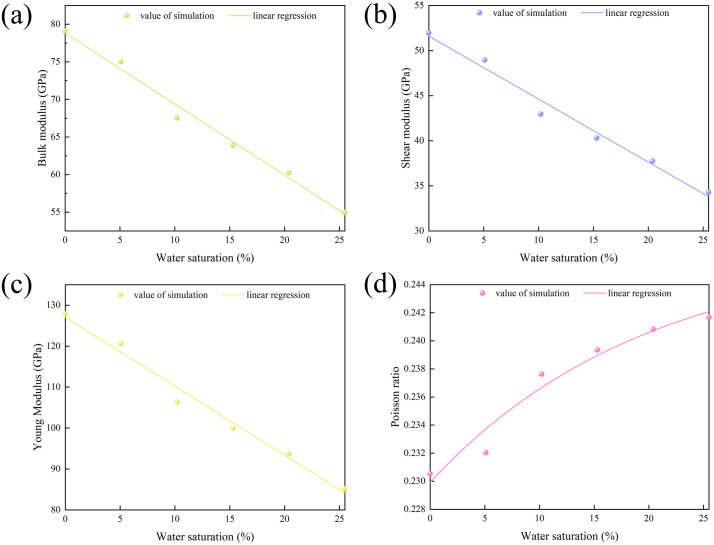
Mechanism of water saturation on mechanical properties. **(a)** bulk modulus; **(b)** Shear modulus; **(c)** Young ‘s modulus; **(d)** Poisson ‘s ratio.

When the water saturation increases from 0% to 25.49%, the decrease ranges of bulk modulus, shear modulus and Young ‘s modulus are 79.100-54.958 GPa, 51.966-34.299 GPa and 127.891-85.178 GPa, respectively, and the decrease ranges are 24.142 GPa, 17.667 GPa and 42.714 GPa, respectively (Figs 13a - 13c). In contrast, the Poisson ‘s ratio increased from 0.230 to 0.242, an increase of 0.012 (Fig 13d).

The increase of saturation will lead to the increase of water pressure in clay minerals and affect their overall mechanical behavior. In the mechanical parameters of hydrated illite, the bulk modulus, shear modulus and Young ‘s modulus decrease with the increase of pressure [26,27]. The decrease of bulk modulus reflects the increase of compressibility of illite. The decrease of shear modulus and Young ‘s modulus indicates that the material is more easily deformed under water lubrication. At the same time, it increases with the increase of water saturation, and the deformation of illite is more significant under transverse stress [28]. On the whole, illite is immersed in water molecules, and the increase of intermolecular force and interlayer water pressure will cause significant expansion effect on its spatial structure. In addition, interlayer water will cause the slip phenomenon of illite crystal layer under shear action, resulting in the overall loss of illite mechanical strength and mechanical deterioration effect.

### Mechanical heterogeneity of hydrated illite

Under the action of hydration, illite has significant scale effect and mechanical heterogeneity. The presence of water molecules seriously affects the mechanical properties of illite [29]. In statistical mechanics, the mechanical parameters such as tensile strength, compressive strength and shear strength of rock and soil have a certain distribution law. The random functions used to reflect the distribution law are usually Weibull function and normal distribution function [30]. Compared with Weibull function, normal distribution function can describe the failure characteristics of rock and soil more accurately in the process of describing stress. The probability density distribution function is calculated as follows:


$$f(x)=\frac{\text{1}}{\sigma \sqrt{\text{2}\pi }}e^{-\frac{x-u}{\text{2}\sigma^{\text{2}}}}$$ (17)

where: u is the expected value, which determines the position of the normal distribution function and reflects the overall performance in heterogeneity; σ is the standard deviation, which determines the amplitude of the function distribution in the function and characterizes the deviation value in the heterogeneity.

In order to further quantify the influence of hydration on the mechanical heterogeneity of illite, the coefficient of variation ${\text{C}}_{\text{v}}$ is introduced to measure the mechanical heterogeneity of water molecules on anhydrous illite. The calculation method of coefficient of variation ${\text{C}}_{\text{v}}$ is as follows [31]:


$$C_{v}=\frac{E_{d}}{E_{m}}$$ (18)

In the formula, $E_{d}$ is the standard deviation; $E_{m\;}$ expected value.

Because water molecules have a great influence on the homogeneity of illite, anhydrous illite with water saturation of 0% can be used as a homogeneous body, and its mechanical parameters can be used as the expected value of calculation. The coefficient of variation ${\text{C}}_{\text{v}}$ is 0 [32].

**Fig 14:**
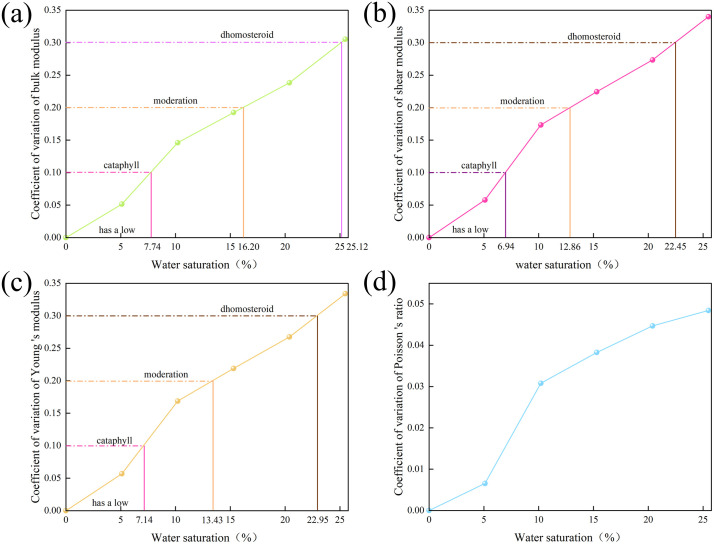
Evolution law of mechanical heterogeneity of hydrated illite. **(a)** Coefficient of variation of bulk modulus; **(b)** Coefficient of variation of shear modulus; **(c)** Young ‘s modulus coefficient of variation; **(d)** Coefficient of variation of Poisson ‘s ratio.

According to the calculation, the mechanical heterogeneity of illite is consistent with the evolution of structural heterogeneity, which increases with the increase of water saturation. With the increase of water saturation from 0% to 25.49%, the variation parameters of illite bulk modulus, shear modulus, Young ‘s modulus and Poisson ‘s ratio increased from 0 to 0.305, 0.340, 0.334 and 0.048, respectively. Based on the evaluation criteria of parameter variability given by the specification, the evolution of the variation properties of various mechanical parameters of hydrated illite is partitioned. The results show that the heterogeneity of bulk modulus, shear modulus and Young ‘s modulus of hydrated illite is divided into four parts:When the water saturation is lower than 7.74%, 6.94% and 7.14% respectively, the variability of bulk modulus, shear modulus and Young ‘s modulus is very low (Fig 14a); When the water saturation is 7.74% − 16.20%, 6.94% − 12.86% and 1.43% − 7.14%, the variability is low (Fig 14b). When the water saturation is between 16.20% −25.12%, 12.86% −22.45% and 1.43% −22.95% respectively, the variability is moderate (Fig 13c). When the water saturation is higher than 25.12%, 22.45% and 22.95%, the variability is high. However, Poisson ‘s ratio is only a part, and its variability is very low (Fig 14d).

At low water saturation, less water molecules are evenly distributed between illite layers. After illite adsorbs water molecules, its interlayer potassium ions are hydrated by water molecules to form an outer sphere complex structure of hydrated potassium ions, which effectively limits the penetration and migration of water molecules, showing high structural homogeneity. At this time, the variability of its mechanical parameters is very low, and the mechanical properties also show high homogeneity. With the continuous increase of water saturation, the limited illite interlayer and potassium ion are difficult to accommodate and adsorb more water molecules, and the illite lamellae are continuously filled with water molecules [33,15]. At this time, the randomness of the combination and distribution of water molecules and potassium ions is enhanced, and the structure appears Strong complexity, mechanical heterogeneity also increases. At high water saturation, water molecules completely penetrate and erode the illite lamellae, and the local hydration state may cause some areas to be more flexible than other areas. This causes the concentration of strain under applied stress and forms obvious heterogeneity. When large deformation occurs in some areas, it may affect the adjacent areas, causing a chain reaction and further enhancing the overall mechanical inhomogeneity [34].

In summary, the mechanical properties of illite are determined by the microstructure of illite crystal (particle distribution, hydration phenomenon, etc.), the interaction force between particles (hydrogen bonding, van der Waals force, etc.), the type of material and its content (inorganic cations represented by potassium ions, water molecules) and other conditions. It can be seen that due to the size effect and structural heterogeneity, illite appears mechanical heterogeneity. Therefore, although the mechanical properties of illite analyzed by micromechanical experiments and molecular dynamics simulation are quite different, they can also be reasonably explained.

## Conclusion

Based on the intermolecular interaction and micromechanical simulation of shale clay minerals under multi-coupling conditions under high pressure, high temperature and water content in shale reservoirs, the effects of temperature, water saturation and pressure on micromechanical properties under the influence of shale reservoir environment are revealed, which provides data support and model verification for shale reservoir drilling and fracturing technology. It provides a direct atomic-level theoretical basis for the prediction of hydration degradation risk of illite formation and the optimization of drilling fluid formulation during drilling.

(1) In the process of water absorption of illite, the main expansion direction is the crystal z direction. Under certain conditions, pressure helps to inhibit the swelling process of illite, thus weakening the infiltration and erosion of illite by water molecules.
(2) Compared with hydrogen atoms in water, oxygen atoms in water are more inclined to potassium ions, and the interaction distance between them is shorter. Pressure will strengthen the interaction between hydrogen and oxygen atoms in water and potassium ions, making them more closely combined, which is not conducive to the diffusion and migration of water molecules. The increase of temperature and water saturation will enhance the diffusivity of water molecules.
(3) Under certain conditions, the elastic modulus of illite increases with the increase of pressure, and decreases with the increase of temperature and water saturation. With the increase of water saturation from 0% to 25.49%, the bulk modulus, shear modulus and Young ‘s modulus of illite show a change of mechanical heterogeneity from low to high, while the mechanical heterogeneity of Poisson ‘s ratio in the range of hydration strength is low.

## Supporting information

S1 File This file is the original data of the experiment and modeling in this paper. It is also the support data of the main graph files in this paper. It can be viewed and uploaded in pdf format.
(PDF)
