# Supplementary material for: Study on hydration characteristics and micromechanical properties of illite based on molecular dynamics
Source: PLoS One. 2026 May 12;21(5):e0346820. doi: 10.1371/journal.pone.0346820 (PMC13166942; doi:10.1371/journal.pone.0346820)
Supplement: S1 File — It is also the support data of the main graph files in this paper. It can be viewed and uploaded in pdf format. (PDF) [file pone.0346820.s001.pdf]

Number of water molecules	Fig. 3 (a)
	Lattice constant
	\f:Times New Roman(Å)
	a
0	20.8084
9	21.19802
18	21.70383
27	22.69814
36	22.85535
45	23.33916

) Illite crystal length parameters under different hydration degrees ;	
Lattice constant	Lattice constant
Å	Å
b	c
17.9594	10.226
18.29568	10.41747
18.73223	10.66605
19.59041	11.15469
19.72609	11.23194
20.14366	11.46971

Fig. 3 (b) The density and volume parameters of illite [illegible]	
Number of water molecules	Volume
\f:Times New Roman()	Å\+(3)
0	3743.87
9	3922.48
18	4413.86
27	4602.5
36	4820.66
45	5079.23

crystals under different hydration degrees
Density
g·cm\+(-3)
2.821
2.73631
2.6128
2.39506
2.29171
2.25406

Fig. 4c The data of the evolution law of the basic interlayer spac		
Pressure(MPa)	Interlayer spacing	Interlayer spacing
MPa	Å	Å
	9H20	18H20
0.101	10.44992	10.72095
100	10.41747	10.71857
200	10.39104	10.66605
300	10.3861	10.6583
400	10.33474	10.6188
500	10.333	10.57271

:ing of illite under the action of pressure on the interlayer spacing		
Interlayer spacing	Interlayer spacing	Interlayer spacing
Å	Å	Å
27H20	36H20	45H20
11.53614	11.28708	11.53614
11.0455	11.27736	11.49317
10.71857	11.23194	11.49063
10.6583	11.22854	11.46971
10.6188	11.17709	11.32048
10.57271	11.15469	11.32

Fig.4a Data of inter		
Number of water molecules	Interlayer spacing	Interlayer spacing
ind	Å	Å
	0.101MPa	100MPa
0	10.226	10.226
9	10.3767	10.41747
18	10.66605	10.6583
27	11.12682	11.12469
36	11.23194	11.22736
45	11.53614	11.53614

·layer spacing evolution of hydrated illite at 25 °C

Interlayer spacing	Interlayer spacing	Interlayer spacing
Å	Å	Å
200MPa	300MPa	400MPa
10.226	10.226	10.226
10.39104	10.3861	10.33474
10.65461	10.6188	10.57271
11.01709	10.80285	10.72095
11.12608	11.02	10.97396
11.49317	11.49063	11.46971

Interlayer spacing
Å
500MPa
10.226
10.333
10.44992
10.71857
10.9455
11.22854

Fig. 4b The data of the evolution law of the interlayer spacing of		
Number of water molecules	Lnterlayer spacing	Lnterlayer spacing
	Å	Å
	25℃	50℃
0	10.226	10.226
9	10.3767	10.39272
18	10.66605	10.7166
27	11.12682	11.15469
36	11.23194	11.29473
45	11.53614	11.43094

hydrated illite under 0.101 MPa	
Lnterlayer spacing	Lnterlayer spacing
Å	Å
100℃	200℃
10.226	10.226
10.404	10.41747
10.72938	10.85309
11.18771	11.21757
11.40488	11.4071
11.44155	11.53614

Fig.4d The evolution law data of illite basic interlayer spaci
Temperature
K
25
50
100
200

ing under the action of temperature on interlayer spacing

Interlayer spacing
Å
45H20
11.53614
11.56971
11.63614
11.69063

Fig. 5a Radial distribution function data of hydration illit		
r	g(r)	g(r)
Å	K\+(+)-O	K\+(+)-O
	101kPa	0.1GPa
0.025	0	0
0.075	0	0
0.125	0	0
0.175	0	0
0.225	0	0
0.275	0	0
0.325	0	0
0.375	0	0
0.425	0	0
0.475	0	0
0.525	0	0
0.575	0	0
0.625	0	0
0.675	0	0
0.725	0	0
0.775	0	0
0.825	0	0
0.875	0	0
0.925	0	0
0.975	0	0
1.025	0	0
1.075	0	0
1.125	0	0
1.175	0	0
1.225	0	0
1.275	0	0
1.325	0	0
1.375	0	0
1.425	0	0
1.475	0	0
1.525	0	0
1.575	0	0
1.625	0	0
1.675	0	0
1.725	0	0
1.775	0	0
1.825	0	0
1.875	0	0
1.925	0	0
1.975	0	0
2.025	0	0
2.075	0	9.09E-4
2.125	0	0.00347
2.175	0	4.13E-4
2.225	0	0
2.275	0	0
2.325	0	0

2.375	0	0
2.425	7.9E-4	3.33E-4
2.475	0.00985	0.03736
2.525	0.13473	0.24023
2.575	0.7633	1.08092
2.625	2.23584	2.8791
2.675	4.42645	5.32518
2.725	6.30994	6.30994
2.775	6.81101	7.17354
2.825	7.07354	7.27186
2.875	6.52679	6.52679
2.925	5.46001	6.06226
2.975	4.43232	4.98476
3.025	3.65121	4.10394
3.075	2.98222	3.30295
3.125	2.52001	2.72578
3.175	2.16697	2.30354
3.225	1.91904	2.00021
3.275	1.74422	1.76287
3.325	1.59094	1.58339
3.375	1.44326	1.44821
3.425	1.38025	1.40874
3.475	1.27718	1.30807
3.525	1.23766	1.30476
3.575	1.16586	1.2604
3.625	1.08056	1.23585
3.675	0.99463	1.21143
3.725	0.96744	1.20831
3.775	0.95176	1.19491
3.825	0.95037	1.12871
3.875	0.97022	1.1494
3.925	1.04301	1.10939
3.975	1.07865	1.12139
4.025	1.15076	1.15806
4.075	1.18799	1.14914
4.125	1.21967	1.18663
4.175	1.24937	1.18273
4.225	1.2244	1.21923
4.275	1.20062	1.23712
4.325	1.17004	1.23995
4.375	1.1198	1.25622
4.425	1.07958	1.24078
4.475	1.07681	1.18361
4.525	1.04883	1.13945
4.575	1.02947	1.09038
4.625	1.02958	1.04005
4.675	0.99387	1.02078
4.725	0.96556	0.98852
4.775	0.91969	0.95951
4.825	0.88058	0.93276
4.875	0.82627	0.925

4.925	0.81752	0.91962
4.975	0.81571	0.92557

e potassium ion and oxygen atom in water with water saturation of 25.39 % at 25 °C.

g(r)	g(r)	g(r)
K\+(+)-O	K\+(+)-O	K\+(+)-O
0.2GPa	0.3GPa	0.4GPa
0	0	0
0	0	0
0	0	0
0	0	0
0	0	0
0	0	0
0	0	0
0	0	0
0	0	0
0	0	0
0	0	0
0	0	0
0	0	0
0	0	0
0	0	0
0	0	0
0	0	0
0	0	0
0	0	0
0	0	0
0	0	0
0	0	0
0	0	0
0	0	0
0	0	0
0	0	0
0	0	0
0	0	0
0	0	0
0	0	0
0	0	0
0	0	0
0	0	0
0	0	0
0	0	0
0	0	0
0	0	0
0	0	0
0	0	0
0	0	0
0	0	0
9.03E-4	8.81E-4	9.12E-4
0.00344	0.00336	0.00348
4.11E-4	4.01E-4	4.15E-4
0	0	0
0	0	0
0	0	0

0	0	6.96E-4
0.00165	0	0.00134
0.01681	0.01455	0.01282
0.1768	0.17076	0.12289
0.80602	0.88788	0.6598
2.26535	2.55955	2.01329
4.23445	4.87333	4.12444
5.85103	6.65214	6.20685
6.75632	7.73037	7.8115
7.49189	7.18461	7.8115
6.30108	6.47406	7.20435
5.49759	5.36855	6.34254
4.61256	4.33157	5.35285
3.88685	3.47407	4.26773
3.21064	2.89525	3.50458
2.72205	2.41665	2.74668
2.31195	2.03126	2.28607
2.08273	1.80154	1.89195
1.84568	1.62865	1.56183
1.6721	1.52754	1.35608
1.55451	1.44299	1.17001
1.43374	1.34748	1.04922
1.3165	1.2438	0.96493
1.26111	1.14497	0.89082
1.18457	1.11569	0.86884
1.19205	1.04947	0.81455
1.16243	0.97434	0.83543
1.17737	0.96627	0.84103
1.18717	0.93299	0.83254
1.21637	0.92729	0.85857
1.27411	0.92082	0.91188
1.29963	0.9363	0.90192
1.32667	0.93702	0.9348
1.36661	0.94374	0.94142
1.36664	0.95407	0.95204
1.37734	0.97489	0.95934
1.38669	0.99227	0.95914
1.37856	0.97997	0.95615
1.35937	0.99153	0.96228
1.33519	1.01994	0.96956
1.34627	1.01579	0.97389
1.30897	1.02522	0.95341
1.28726	1.04487	0.96703
1.24483	1.04154	0.95105
1.20458	1.06131	0.96669
1.16342	1.07848	0.99437
1.15031	1.0938	0.98705
1.1281	1.12617	1.02027
1.10809	1.15012	1.08359
1.08049	1.17521	1.10637
1.07283	1.17653	1.14023

1.03946	1.18263	1.17419
1.02621	1.18427	1.18744

g(r)
K\+(+)-O
0.5GPa
0
0
0
0
0
0
0
0
0
0
0
0
0
0
0
0
0
0
0
0
0
0
0
0
0
0
0
0
0
0
0
0
0
0
0
0
0
0
0
0
0
8.62E-4
0.00329
3.92E-4
0
0
0

3.29E-4
0.0041
0.03788
0.31213
1.30158
3.30508
5.54816
7.2484
8.26809
7.19072
6.0141
4.88354
3.90674
3.07146
2.53105
2.0842
1.82148
1.61408
1.4878
1.34802
1.2717
1.2513
1.20971
1.16697
1.1379
1.07508
1.07627
1.01305
0.95617
0.89048
0.84589
0.78567
0.77449
0.78092
0.76367
0.76447
0.80708
0.85974
0.91949
0.96366
1.01876
1.08044
1.11159
1.16078
1.20632
1.21735
1.24751
1.23048
1.25402
1.24109
1.21763

1.19112
1.19243

Fig. 5a Radial distribution function of hydrous illite potassium ion with water saturatio			
r	g(r)	g(r)	g(r)
Å	K\+(+)-H	K\+(+)-H	K\+(+)-H
	101kPa	0.1GPa	0.2GPa
0.025	0	0	0
0.075	0	0	0
0.125	0	0	0
0.175	0	0	0
0.225	0	0	0
0.275	0	0	0
0.325	0	0	0
0.375	0	0	0
0.425	0	0	0
0.475	0	0	0
0.525	0	0	0
0.575	0	0	0
0.625	0	0	0
0.675	0	0	0
0.725	0	0	0
0.775	0	0	0
0.825	0	0	0
0.875	0	0	0
0.925	0	0	0
0.975	0	0	0
1.025	0	0	0
1.075	0	0	0
1.125	0	0	0
1.175	0	0	0
1.225	0	0	0
1.275	0	0	0
1.325	0	0	0
1.375	0	0	0
1.425	0	0	0
1.475	0	0	0
1.525	0	0	0
1.575	0	0	0
1.625	0	0	0
1.675	0	0	0
1.725	0	0	0
1.775	0	0	0
1.825	0	0	0
1.875	0	0	0
1.925	0	0	0
1.975	0	0	0
2.025	0	0	0
2.075	0	0	0
2.125	0	0	0
2.175	7.28E-4	4.15E-4	6.16E-4
2.225	0.00186	9.92E-4	0.00255
2.275	0.01109	0.00664	0.01014
2.325	0.02995	0.0267	0.02589

2.375	0.07735	0.07554	0.06701
2.425	0.20307	0.17513	0.15367
2.475	0.42307	0.34091	0.2868
2.525	0.69085	0.60411	0.49869
2.575	1.10397	0.87746	0.75268
2.625	1.48024	1.21937	1.05257
2.675	1.833	1.5396	1.36558
2.725	2.09912	1.8303	1.62476
2.775	2.26334	2.07756	1.88688
2.825	2.39414	2.2918	2.09951
2.875	2.46162	2.48705	2.27235
2.925	2.4877	2.58166	2.46883
2.975	2.56565	2.70405	2.61644
3.025	2.7039	2.80037	2.81048
3.075	2.85178	2.88168	2.95568
3.125	2.92916	2.93679	3.14833
3.175	3.08674	3.04741	3.26299
3.225	3.15175	3.11725	3.30003
3.275	3.25997	3.19772	3.34971
3.325	3.21958	3.20947	3.3348
3.375	3.18407	3.21877	3.30566
3.425	3.10823	3.11276	3.2593
3.475	3.01173	3.01627	3.18228
3.525	2.92468	2.8929	3.11134
3.575	2.74174	2.76992	2.99671
3.625	2.58047	2.60482	2.83941
3.675	2.377	2.47576	2.66496
3.725	2.15895	2.26306	2.46714
3.775	1.90102	2.05153	2.25289
3.825	1.70206	1.85222	2.05271
3.875	1.54417	1.60275	1.85272
3.925	1.39246	1.43771	1.68925
3.975	1.25983	1.25388	1.53965
4.025	1.15594	1.12068	1.42629
4.075	1.07747	1.04114	1.3303
4.125	1.00994	0.94537	1.24913
4.175	0.9631	0.89515	1.18522
4.225	0.92127	0.87805	1.12037
4.275	0.88414	0.83818	1.06752
4.325	0.87137	0.81986	1.02854
4.375	0.84959	0.83042	1.01278
4.425	0.82827	0.83472	0.98798
4.475	0.81256	0.84564	0.97651
4.525	0.81163	0.84906	0.96805
4.575	0.8071	0.86827	0.96205
4.625	0.78379	0.88026	0.95903
4.675	0.80604	0.91504	0.9409
4.725	0.80198	0.93118	0.95713
4.775	0.80104	0.94618	0.95509
4.825	0.81994	0.97344	0.94759
4.875	0.84514	0.99258	0.95949

4.925	0.86187	1.00969	0.95629
4.975	0.88087	1.02861	0.95452

n of 25.39 % and hydrogen atom in water at 25 °C		
g(r)	g(r)	g(r)
K\+(+)-H	K\+(+)-H	K\+(+)-H
0.3GPa	0.4GPa	0.5GPa
0	0	0
0	0	0
0	0	0
0	0	0
0	0	0
0	0	0
0	0	0
0	0	0
0	0	0
0	0	0
0	0	0
0	0	0
0	0	0
0	0	0
0	0	0
0	0	0
0	0	0
0	0	0
0	0	0
0	0	0
0	0	0
0	0	0
0	0	0
0	0	0
0	0	0
0	0	0
0	0	0
0	0	0
0	0	0
0	0	0
0	0	0
0	0	0
0	0	0
0	0	0
0	0	0
0	0	0
0	0	0
0	0	0
0	0	0
0	0	0
0	0	0
0	0	0
0	0	0
0	2E-4	4.13E-4
9.38E-4	7.66E-4	0.00257
0.00583	0.00715	0.00416
0.02169	0.01684	0.01484

0.05906	0.05414	0.04213
0.14232	0.12707	0.10445
0.25829	0.23856	0.22353
0.44735	0.43745	0.36311
0.66841	0.67435	0.56258
0.88831	0.94383	0.78209
1.17223	1.18692	0.9792
1.44132	1.39689	1.28052
1.68124	1.67409	1.53682
1.90255	1.88913	1.80718
2.10364	2.15833	2.16044
2.28362	2.47146	2.49818
2.47415	2.75006	2.85418
2.65665	3.1074	3.21765
2.90267	3.35027	3.56557
3.09676	3.52379	3.8501
3.30228	3.69591	4.02853
3.52223	3.762	4.0432
3.61789	3.68373	4.07799
3.71622	3.58888	3.99227
3.67715	3.45277	3.92535
3.66448	3.23588	3.77048
3.48606	3.09294	3.585
3.26885	2.89493	3.3347
3.02473	2.67161	3.11282
2.72729	2.45545	2.84377
2.40545	2.23152	2.56342
2.12396	2.00539	2.35748
1.89431	1.81793	2.08604
1.67199	1.65125	1.87002
1.47257	1.51304	1.6761
1.32189	1.3893	1.52713
1.18048	1.28357	1.38589
1.08727	1.21248	1.29227
1.00819	1.14419	1.21287
0.95285	1.09382	1.14105
0.90926	1.04624	1.12797
0.88981	1.02215	1.09036
0.87554	0.98842	1.05355
0.89258	0.9769	1.02374
0.89304	0.95896	0.99454
0.92651	0.9517	0.98169
0.94441	0.94054	0.94688
0.98488	0.9271	0.94246
1.02248	0.93594	0.9301
1.04315	0.93249	0.91074
1.06276	0.91902	0.91239
1.07501	0.93098	0.91294
1.05903	0.93447	0.92777
1.04936	0.94587	0.90868
1.02927	0.9533	0.93121

1.00757	0.97271	0.92333
1.00135	0.96912	0.93584

Fig.6 (a) Radial distribution function data of potassium ions and oxygen atoms in water v		
r	g(r)	g(r)
Å	K\+(+)-O	K\+(+)-O
	25℃	50℃
0.025	0	0
0.075	0	0
0.125	0	0
0.175	0	0
0.225	0	0
0.275	0	0
0.325	0	0
0.375	0	0
0.425	0	0
0.475	0	0
0.525	0	0
0.575	0	0
0.625	0	0
0.675	0	0
0.725	0	0
0.775	0	0
0.825	0	0
0.875	0	0
0.925	0	0
0.975	0	0
1.025	0	0
1.075	0	0
1.125	0	0
1.175	0	0
1.225	0	0
1.275	0	0
1.325	0	0
1.375	0	0
1.425	0	0
1.475	0	0
1.525	0	0
1.575	0	0
1.625	0	0
1.675	0	0
1.725	0	0
1.775	0	0
1.825	0	0
1.875	0	0
1.925	0	0
1.975	0	0
2.025	0	0
2.075	0	0
2.125	0	0
2.175	0	0
2.225	0	0
2.275	0	0
2.325	0	0

2.375	0	0
2.425	7.9E-4	0.00117
2.475	0.00985	0.03265
2.525	0.13473	0.29062
2.575	0.7633	1.11673
2.625	2.23584	2.714
2.675	4.42645	3.23803
2.725	6.30994	4.89871
2.775	6.81101	6.00338
2.825	7.07354	6.40217
2.875	6.52679	6.14129
2.925	5.46001	5.56313
2.975	4.43232	4.78109
3.025	3.65121	3.84023
3.075	2.98222	3.36626
3.125	2.52001	2.89571
3.175	2.16697	2.52404
3.225	1.91904	2.2658
3.275	1.74422	1.99524
3.325	1.59094	1.77225
3.375	1.44326	1.63819
3.425	1.38025	1.56562
3.475	1.27718	1.47711
3.525	1.23766	1.43088
3.575	1.16586	1.45193
3.625	1.08056	1.43192
3.675	0.99463	1.37484
3.725	0.96744	1.3468
3.775	0.95176	1.34475
3.825	0.95037	1.32145
3.875	0.97022	1.27991
3.925	1.04301	1.24736
3.975	1.07865	1.2527
4.025	1.15076	1.24178
4.075	1.18799	1.17121
4.125	1.21967	1.17014
4.175	1.24937	1.12606
4.225	1.2244	1.08167
4.275	1.20062	1.04066
4.325	1.17004	1.05226
4.375	1.1198	1.0251
4.425	1.07958	1.0603
4.475	1.07681	1.05304
4.525	1.04883	1.09052
4.575	1.02947	1.09461
4.625	1.02958	1.07676
4.675	0.99387	1.08783
4.725	0.96556	1.04341
4.775	0.91969	1.04254
4.825	0.88058	1.01828
4.875	0.82627	0.99586

4.925	0.81752	0.99299
4.975	0.81571	0.96282

vith water saturation of 25.39 % at 0.101 MPa.

g(r)	g(r)
K\+(+)-O	K\+(+)-O
100℃	200℃
0	0
0	0
0	0
0	0
0	0
0	0
0	0
0	0
0	0
0	0
0	0
0	0
0	0
0	0
0	0
0	0
0	0
0	0
0	0
0	0
0	0
0	0
0	0
0	0
0	0
0	0
0	0
0	0
0	0
0	0
0	0
0	0
0	0
0	0
0	0
0	0
0	0
0	0
0	0
0	0
0	0
0	0
0	0
0	0
0	0
0	0
0	0

4.11E-4	0
3.94E-4	0.00118
0.02268	0.01548
0.17397	0.15197
0.94326	0.72331
2.7331	1.59177
3.85273	3.10241
5.30689	4.5948
5.99315	5.39847
5.99766	5.62698
5.51265	5.26958
4.87422	5.13352
4.16334	4.2426
3.57998	3.6408
3.00435	3.06042
2.64436	2.62749
2.2677	2.33549
1.99377	2.07242
1.79065	1.87358
1.61362	1.70868
1.45639	1.57154
1.36167	1.49425
1.29592	1.41422
1.25289	1.32042
1.2161	1.28989
1.17256	1.21443
1.17019	1.18504
1.1218	1.13394
1.14412	1.12714
1.10744	1.12142
1.08537	1.06448
1.03835	1.10418
1.02588	1.0776
1.00669	1.08911
0.9675	1.07383
0.94065	1.06997
0.90364	1.06638
0.89717	1.06772
0.91786	1.04011
0.9136	1.01706
0.91848	1.05073
0.95142	1.00917
0.95768	0.98559
0.98549	0.97918
0.99759	0.97369
1.0216	0.97459
1.01248	0.9875
1.00548	1.00504
0.9957	1.02842
1.01396	1.05023
0.99649	1.06811

0.98953	1.10802
0.97395	1.12109

ig.6 (b) Radial distribution function data of potassium ions and hydrogen atoms in wat		
r	g(r)	g(r)
Å	K\+(+)-H	K\+(+)-H
	25℃	50℃
0.025	0	0
0.075	0	0
0.125	0	0
0.175	0	0
0.225	0	0
0.275	0	0
0.325	0	0
0.375	0	0
0.425	0	0
0.475	0	0
0.525	0	0
0.575	0	0
0.625	0	0
0.675	0	0
0.725	0	0
0.775	0	0
0.825	0	0
0.875	0	0
0.925	0	0
0.975	0	0
1.025	0	0
1.075	0	0
1.125	0	0
1.175	0	0
1.225	0	0
1.275	0	0
1.325	0	0
1.375	0	0
1.425	0	0
1.475	0	0
1.525	0	0
1.575	0	0
1.625	0	0
1.675	0	0
1.725	0	0
1.775	0	0
1.825	0	0
1.875	0	0
1.925	0	0
1.975	0	0
2.025	0	0
2.075	0	2.73E-4
2.125	0	7.81E-4
2.175	7.28E-4	7.45E-4
2.225	0.00186	0.00427
2.275	0.01109	0.01113
2.325	0.02995	0.03109

2.375	0.07735	0.08501
2.425	0.20307	0.18487
2.475	0.42307	0.32982
2.525	0.69085	0.54511
2.575	1.10397	0.80811
2.625	1.48024	1.09488
2.675	1.833	1.39138
2.725	2.09912	1.65323
2.775	2.26334	1.90769
2.825	2.39414	2.11704
2.875	2.46162	2.28166
2.925	2.4877	2.37068
2.975	2.56565	2.52911
3.025	2.7039	2.61561
3.075	2.85178	2.6988
3.125	2.92916	2.83494
3.175	3.08674	2.90784
3.225	3.15175	2.94087
3.275	3.25997	3.03706
3.325	3.21958	3.04943
3.375	3.18407	3.08821
3.425	3.10823	3.09158
3.475	3.01173	3.04608
3.525	2.92468	2.98015
3.575	2.74174	2.90666
3.625	2.58047	2.79197
3.675	2.377	2.61174
3.725	2.15895	2.38759
3.775	1.90102	2.15833
3.825	1.70206	1.95068
3.875	1.54417	1.75906
3.925	1.39246	1.55859
3.975	1.25983	1.38871
4.025	1.15594	1.25924
4.075	1.07747	1.15711
4.125	1.00994	1.05698
4.175	0.9631	1.00814
4.225	0.92127	0.94637
4.275	0.88414	0.88256
4.325	0.87137	0.84065
4.375	0.84959	0.81983
4.425	0.82827	0.80615
4.475	0.81256	0.80168
4.525	0.81163	0.81448
4.575	0.8071	0.81121
4.625	0.78379	0.81673
4.675	0.80604	0.82016
4.725	0.80198	0.8367
4.775	0.80104	0.8305
4.825	0.81994	0.84029
4.875	0.84514	0.86439

4.925	0.86187	0.87944
4.975	0.88087	0.90464

er with water saturation of 25.39 % at 0.101 MP[illegible]

g(r)	g(r)
K\+(+)-H	K\+(+)-H
100℃	200℃
0	0
0	0
0	0
0	0
0	0
0	0
0	0
0	0
0	0
0	0
0	0
0	0
0	0
0	0
0	0
0	0
0	0
0	0
0	0
0	0
0	0
0	0
0	0
0	0
0	0
0	0
0	0
0	0
0	0
0	0
0	0
0	0
0	0
0	0
0	0
0	0
0	0
0	0
0	0
0	0
0	0
2.16E-4	0
0	7.59E-4
5.89E-4	0.00121
0.00187	0.00554
0.00592	0.01743
0.01236	0.04078

0.04213	0.09051
0.09991	0.17402
0.1991	0.28528
0.38318	0.45359
0.65317	0.63975
0.93444	0.86209
1.26122	1.03639
1.56948	1.25065
1.83056	1.44019
2.04675	1.64384
2.25164	1.82442
2.34728	1.98699
2.47438	2.15267
2.57585	2.25784
2.70057	2.37224
2.78042	2.44875
2.84096	2.54977
2.96765	2.63682
3.02207	2.75734
2.99836	2.7706
3.00935	2.77014
2.96921	2.75888
2.88	2.74182
2.78073	2.63316
2.62273	2.54706
2.41759	2.3283
2.16339	2.17413
1.94221	1.93836
1.77067	1.7398
1.60218	1.54573
1.47796	1.41703
1.38595	1.31028
1.33192	1.23907
1.25458	1.2003
1.18295	1.16972
1.12564	1.13186
1.07733	1.12294
1.02588	1.0802
1.00929	1.07702
0.96852	1.04524
0.95228	1.01569
0.94856	0.98704
0.93597	0.95877
0.96621	0.93636
0.97133	0.89325
0.98268	0.88734
1.00526	0.8822
0.99853	0.87995
1.01266	0.86403
1.00832	0.88472
1.00431	0.90291

1.02611	0.89206
1.01733	0.90117

Fig.7 (a) Radial distribution function data of potassium ions and oxygen ator

r	g(r)	g(r)
Å	K\+(+)-O	K\+(+)-O
	25.49%	20.39%
0.025	0	0
0.075	0	0
0.125	0	0
0.175	0	0
0.225	0	0
0.275	0	0
0.325	0	0
0.375	0	0
0.425	0	0
0.475	0	0
0.525	0	0
0.575	0	0
0.625	0	0
0.675	0	0
0.725	0	0
0.775	0	0
0.825	0	0
0.875	0	0
0.925	0	0
0.975	0	0
1.025	0	0
1.075	0	0
1.125	0	0
1.175	0	0
1.225	0	0
1.275	0	0
1.325	0	0
1.375	0	0
1.425	0	0
1.475	0	0
1.525	0	0
1.575	0	0
1.625	0	0
1.675	0	0
1.725	0	0
1.775	0	0
1.825	0	0
1.875	0	0
1.925	0	0
1.975	0	0
2.025	0	0
2.075	0	0
2.125	0	0
2.175	0	0
2.225	0	0
2.275	0	0
2.325	0	0

2.375	0	0
2.425	7.9E-4	0.00227
2.475	0.00985	0.10744
2.525	0.13473	0.73645
2.575	0.7633	2.34591
2.625	2.23584	4.5439
2.675	4.42645	6.567
2.725	6.30994	7.55971
2.775	7.27186	7.27099
2.825	7.17354	6.25993
2.875	6.52679	5.11934
2.925	5.46001	4.05897
2.975	4.43232	3.24475
3.025	3.65121	2.64543
3.075	2.98222	2.27913
3.125	2.52001	1.98926
3.175	2.16697	1.80554
3.225	1.91904	1.77417
3.275	1.74422	1.6371
3.325	1.59094	1.63494
3.375	1.44326	1.63148
3.425	1.38025	1.50437
3.475	1.27718	1.40445
3.525	1.23766	1.35564
3.575	1.16586	1.33598
3.625	1.08056	1.25826
3.675	0.99463	1.29596
3.725	0.96744	1.26372
3.775	0.95176	1.29167
3.825	0.95037	1.33401
3.875	0.97022	1.38819
3.925	1.04301	1.36503
3.975	1.07865	1.35105
4.025	1.15076	1.35783
4.075	1.18799	1.35097
4.125	1.21967	1.29578
4.175	1.24937	1.24912
4.225	1.2244	1.2494
4.275	1.20062	1.20966
4.325	1.17004	1.22419
4.375	1.1198	1.2158
4.425	1.07958	1.25953
4.475	1.07681	1.29046
4.525	1.04883	1.29647
4.575	1.02947	1.30395
4.625	1.02958	1.29154
4.675	0.99387	1.26357
4.725	0.96556	1.25914
4.775	0.91969	1.19759
4.825	0.88058	1.17416
4.875	0.82627	1.15729

4.925	0.81752	1.15322
4.975	0.81571	1.17772

ns in water with different water saturations at 25 °C and 0.101 MPa

g(r)	g(r)	g(r)
K\+(+)-O	K\+(+)-O	K\+(+)-O
15.30%	10.20%	5.10%
0	0	0
0	0	0
0	0	0
0	0	0
0	0	0
0	0	0
0	0	0
0	0	0
0	0	0
0	0	0
0	0	0
0	0	0
0	0	0
0	0	0
0	0	0
0	0	0
0	0	0
0	0	0
0	0	0
0	0	0
0	0	0
0	0	0
0	0	0
0	0	0
0	0	0
0	0	0
0	0	0
0	0	0
0	0	0
0	0	0
0	0	0
0	0	0
0	0	0
0	0	0
0	0	0
0	0	0
0	0	0
0	0	0
0	0	0
0	0	0
0	0	0
0	0	0
0	0	0
0	0	0
0	0	0
0	0	0
0	0	0

4.95E-4	0.00582	5.94E-4
0	0.13245	0.00798
0.00912	1.21254	0.1488
0.10641	4.44582	0.99921
0.57012	7.76918	3.52927
1.86501	8.66019	7.08791
4.2341	7.71523	9.74592
6.50561	6.73774	9.79502
7.82286	6.00968	8.36555
7.84366	5.65185	6.59017
6.77269	5.11127	5.22528
5.47638	4.50258	3.9949
4.42763	3.91354	3.21731
3.52306	3.38528	2.70898
2.88681	2.78681	2.38804
2.47326	2.35433	2.31052
2.18188	2.05408	2.20774
1.88497	1.7414	2.14497
1.69744	1.52371	2.08529
1.50864	1.41712	1.98576
1.43486	1.2498	1.87617
1.42921	1.22278	1.75123
1.3893	1.20727	1.54384
1.39511	1.19064	1.51897
1.39939	1.21153	1.45003
1.43967	1.28046	1.47865
1.44066	1.25976	1.48633
1.494	1.3709	1.45346
1.49818	1.40002	1.45802
1.51537	1.40086	1.45474
1.50571	1.3215	1.42502
1.42283	1.29688	1.43354
1.41111	1.18933	1.38625
1.3163	1.11594	1.38388
1.30572	1.01639	1.33076
1.26441	0.95771	1.31268
1.20852	0.87514	1.2647
1.19729	0.75204	1.20152
1.14638	0.66086	1.08556
1.13226	0.59325	0.98429
1.09793	0.51676	0.93986
1.11589	0.52696	0.8792
1.13473	0.5532	0.8933
1.11838	0.59444	0.91655
1.12969	0.61044	0.9642
1.09991	0.63394	0.98216
1.08724	0.66232	0.9835
1.09499	0.69756	0.99251
1.12655	0.72171	1.01431
1.1297	0.77778	0.99887
1.12486	0.84779	1.01473

1.12861	0.91752	1.01399
1.1031	0.95131	1.00264

Fig.7 (b) Radial distribution function data of potassium ions and hydrogen atoms in water witl			
r	g(r)	g(r)	g(r)
Å	K\+(+)-H	K\+(+)-H	K\+(+)-H
	25.49%	20.39%	15.30%
0.025	0	0	0
0.075	0	0	0
0.125	0	0	0
0.175	0	0	0
0.225	0	0	0
0.275	0	0	0
0.325	0	0	0
0.375	0	0	0
0.425	0	0	0
0.475	0	0	0
0.525	0	0	0
0.575	0	0	0
0.625	0	0	0
0.675	0	0	0
0.725	0	0	0
0.775	0	0	0
0.825	0	0	0
0.875	0	0	0
0.925	0	0	0
0.975	0	0	0
1.025	0	0	0
1.075	0	0	0
1.125	0	0	0
1.175	0	0	0
1.225	0	0	0
1.275	0	0	0
1.325	0	0	0
1.375	0	0	0
1.425	0	0	0
1.475	0	0	0
1.525	0	0	0
1.575	0	0	0
1.625	0	0	0
1.675	0	0	0
1.725	0	0	0
1.775	0	0	0
1.825	0	0	0
1.875	0	0	0
1.925	0	0	0
1.975	0	0	0
2.025	0	0	0
2.075	0	0	0
2.125	0	0	0
2.175	7.28E-4	0	2.85E-4
2.225	0.00186	9.38E-4	0.00136
2.275	0.01109	0.00583	0.00651
2.325	0.02995	0.02169	0.02343

2.375	0.07735	0.05906	0.05949
2.425	0.20307	0.14232	0.15972
2.475	0.42307	0.25829	0.32822
2.525	0.69085	0.44735	0.5878
2.575	1.10397	0.66841	0.8989
2.625	1.48024	0.88831	1.24967
2.675	1.833	1.17223	1.53823
2.725	2.09912	1.44132	1.81258
2.775	2.26334	1.68124	2.00317
2.825	2.39414	1.90255	2.22215
2.875	2.46162	2.10364	2.47942
2.925	2.4877	2.28362	2.72205
2.975	2.56565	2.47415	3.03709
3.025	2.7039	2.65665	3.28419
3.075	2.85178	2.90267	3.5307
3.125	2.92916	3.09676	3.70524
3.175	3.08674	3.30228	3.93609
3.225	3.15175	3.52223	4.02152
3.275	3.25997	3.61789	4.09027
3.325	3.21958	3.71622	4.0635
3.375	3.18407	3.67715	4.03473
3.425	3.10823	3.66448	3.76282
3.475	3.01173	3.48606	3.58546
3.525	2.92468	3.26885	3.33892
3.575	2.74174	3.02473	3.15413
3.625	2.58047	2.72729	2.89718
3.675	2.377	2.40545	2.70124
3.725	2.15895	2.12396	2.50587
3.775	1.90102	1.89431	2.26376
3.825	1.70206	1.67199	2.06082
3.875	1.54417	1.47257	1.86986
3.925	1.39246	1.32189	1.69
3.975	1.25983	1.18048	1.56826
4.025	1.15594	1.08727	1.45568
4.075	1.07747	1.00819	1.35233
4.125	1.00994	0.95285	1.29179
4.175	0.9631	0.90926	1.20862
4.225	0.92127	0.88981	1.1527
4.275	0.88414	0.87554	1.10495
4.325	0.87137	0.89258	1.06003
4.375	0.84959	0.89304	1.02383
4.425	0.82827	0.92651	0.97247
4.475	0.81256	0.94441	0.91937
4.525	0.81163	0.98488	0.88534
4.575	0.8071	1.02248	0.86178
4.625	0.78379	1.04315	0.82649
4.675	0.80604	1.06276	0.81248
4.725	0.80198	1.07501	0.79761
4.775	0.80104	1.05903	0.77798
4.825	0.81994	1.04936	0.78388
4.875	0.84514	1.02927	0.77712

4.925	0.86187	1.00757	0.81171
4.975	0.88087	1.00135	0.81796

ı different water saturations at 25 °C and 0.101 MPa	
g(r)	g(r)
K\+(+)-H	K\+(+)-H
10.20%	5.10%
0	0
0	0
0	0
0	0
0	0
0	0
0	0
0	0
0	0
0	0
0	0
0	0
0	0
0	0
0	0
0	0
0	0
0	0
0	0
0	0
0	0
0	0
0	0
0	0
0	0
0	0
0	0
0	0
0	0
0	0
0	0
0	0
0	0
0	0
0	0
0	0
0	0
0	0
0	0
0	0
0	0
0	0
7.06E-4	0
6.74E-4	3.59E-4
0.00805	0.00274
0.02464	0.01345
0.07195	0.04618

0.17634	0.11893
0.40334	0.24374
0.76193	0.44857
1.19859	0.65687
1.57921	0.89106
1.85205	1.12386
2.01647	1.3089
2.09299	1.4756
2.24285	1.75085
2.49073	2.06204
2.75623	2.48652
3.1148	2.95267
3.42829	3.52432
3.78083	3.99079
3.99166	4.36317
4.19108	4.62535
4.29842	4.66091
4.32894	4.75836
4.34193	4.63587
4.2628	4.46662
4.03975	4.16246
3.7543	3.73996
3.30079	3.25887
2.91558	2.73299
2.52254	2.30189
2.21094	2.01628
1.95998	1.73381
1.75814	1.55698
1.58403	1.40291
1.42456	1.2663
1.3391	1.2025
1.20866	1.13723
1.09148	1.0672
1.00697	1.03467
0.90907	1.0216
0.86796	0.97044
0.81675	0.96331
0.80263	0.95577
0.80577	0.94358
0.82542	0.95176
0.84897	0.96926
0.87166	0.97411
0.87505	1.00437
0.87855	1.01837
0.85991	1.03672
0.84261	1.05492
0.82272	1.04903
0.79733	1.03661
0.78848	1.00034
0.76674	0.95403
0.77464	0.92342

0.80066	0.86821
0.80989	0.83562

Fig. 8 K + hydratio	
Water saturation	Hydration parameters of illite
	ind
	Coordination number CN
0.051	3.93197
0.102	3.71032
0.153	3.56867
0.2039	3.39723
0.2549	2.97925

[illegible]n parameter data of illite interlayer	
Hydration parameters of illite	Hydration parameters of illite
ind	ind
Hydration ion number	Hydrated radius
1.57279	3.14322
1.48413	3.14382
1.42747	3.12193
1.35889	3.09819
1.1917	3.08352

Fig. 10 Mechanism data of temperature effect on mechanical properties			
Temperature	Poisson ratio	Bulk modulus	Shear modulus
℃		GPa	GPa
25	0.24169	54.9582	34.299
50	0.26134	51.5853	29.2817
100	0.27084	47.4581	25.6727
200	0.28116	42.3385	21.6956

Young Modulus
GPa
85.17746
73.86832
65.25197
55.59123

Fig. 11 Mechanism data of pressure effect on mechanical properties			
Pressure	Poisson ratio	Bulk modulus	Shear modulus
MPa		GPa	GPa
0.101	0.24169	54.9582	34.299
100	0.23733	62.8934	40.0542
200	0.23484	67.4413	43.445
300	0.22279	71.0979	48.3551
400	0.21153	73.7119	51.2249
500	0.20489	77.8195	57.1805

Young Modulus
GPa
85.17746
99.12065
107.29545
118.2559
124.77195
137.79232

Fig. 12 Mechanism data of water saturation on mechanica		
Water saturation	Poisson ratio	Bulk modulus
%		GPa
0	0.23053	79.1
5.1	0.23204	75.0276
10.2	0.23763	67.5414
15.3	0.23935	63.8724
20.39	0.24083	60.2519
25.49	0.24169	54.9582

l properties	
Shear modulus	Young Modulus
GPa	GPa
51.9661	127.89147
48.9543	120.62714
42.9543	106.3234
40.2988	99.88888
37.7545	93.69367
34.299	85.17746

Water saturation
%
0
5.1
10.2
15.3
20.39
25.49

Coefficient of variation of Poisson 's ratio
0
0.00655
0.0308
0.03826
0.04468
0.04841

Fig.13 Data of evolution law of mechanical heter[illegible]

Coefficient of variation of bulk modulus
0
0.05148
0.14613
0.19251
0.23828
0.30521

ogeneity of hydrated illite

Coefficient of variation of shear modulus
0
0.05796
0.17342
0.22452
0.27348
0.33997

Coefficient of variation of Young 's modulus
0
0.0568
0.16864
0.21896
0.2674
0.33399
